# One-stage jaw reconstruction and prosthetic rehabilitation with an iliac flap: a case report and literature review

**DOI:** 10.1186/s40902-024-00413-0

**Published:** 2024-01-17

**Authors:** Yi-Fan Kang, Yan-Jun Ge, Xiao-Ming Lv, Meng-Kun Ding, Xiao-Feng Shan, Zhi-Gang Cai

**Affiliations:** 1 Department of Oral and Maxillofacial Surgery, Peking University School and Hospital of Stomatology, Beijing, 100081 China; 2 National Engineering Laboratory for Digital and Material Technology of Stomatology, Beijing Key Laboratory of Digital Stomatology, National Center of Stomatology, National Clinical Research Center for Oral Diseases, Research Center of Engineering and Technology for Computerized Dentistry Ministry of Health, National Medical Products Administration (NMPA) Key Laboratory for Dental Materials, , Beijing, China; 3 Department of Prosthodontics, Peking University School and Hospital of Stomatology, Beijing, China

**Keywords:** Jaw reconstruction, Iliac flap, Implant, Immediate dental rehabilitation, Jaw in a day

## Abstract

**Background:**

One-stage jaw reconstruction with fibular flap and prosthetic rehabilitation restores bony and dental continuity simultaneously. It was also called as “jaw-in-a-day (JIAD)” technique. However, bone volume and height of fibular flap may be insufficient for dental implant insertion. The provision of a considerable amount of bone makes an iliac flap the ideal choice in these cases. We present the first case report to document the use of one-stage jaw reconstruction and prosthetic rehabilitation with the iliac flap.

**Case presentation:**

We modified the conventional JIAD workflow to make it suitable for iliac flap. Two cases were presented who both underwent segmental mandibulectomy for ameloblastoma. Virtual surgical planning was performed in all cases. The iliac crest was positioned upward to provide cortical bone for achieving primary stability of dental implants. Similar to the “all-on-4” procedure, the iliac bone was placed 12 to 15 mm below the occlusal plane to create adequate space for the implant-retained prosthesis. Immediate implant-based dental rehabilitation was performed at same stage. The surgery was successful in all cases without any short-term complications. In the first postoperative week, patients were given a liquid diet through a nasal feeding tube. The liquid diet is advised until 1 month after the surgery. Thereafter, a soft diet is recommended. Patients were advised to resume routine mastication and normal diet 3 months after the surgery. Peri-implantitis occurred in one patient, and additional gingival graft was required. Postoperative function and esthetics were satisfactory at the last follow-up visit.

**Conclusions:**

One-stage jaw reconstruction and prosthetic rehabilitation with the iliac flap are safe and useful for restoring postoperative function and esthetics. It should be used in more cases with a longer follow-up in further studies.

**Supplementary Information:**

The online version contains supplementary material available at 10.1186/s40902-024-00413-0.

## Background

Jaw reconstruction is one of the most challenging procedures in oral and maxillofacial surgery, as both esthetics and function need to be restored. The vascularized bone flap has been the golden standard in jaw reconstruction for a decade. The fibular and iliac flaps are commonly used for jaw reconstruction [1, 2]. Removable dental prostheses and implant-supported prostheses were used to recover masticatory function after jaw reconstruction. However, removable dental rehabilitation is challenging since the reconstructed bone with the attached soft tissues lacks keratinization, resulting in an unstable and nonfunctional prosthesis [3]. Dental implants are therefore widely used nowadays for jaw reconstruction, with promising results [4].

In some difficult cases, a poor intermaxillary jaw relationship might cause the failure of prosthetic rehabilitation, requiring a greater number of surgeries and time for correction [5]. Occlusion-driven jaw reconstruction has become popular in recent years [6]. With the help of virtual surgical planning (VSP), the success rate of treatment with dental implants has improved significantly [7]. “Jaw-in-a-day (JIAD),” a typical method of occlusion-driven jaw reconstruction, is one of the most advanced techniques in jaw reconstruction, first reported by Levine et al. [8] Herein, both jaw reconstruction and prosthetic rehabilitation using dental implants are completed at the stage of tumor resection in a single day. Thus, the use of the JIAD technique can reduce the “edentulous period” to zero. VSP and surgical guides play an important role in the implementation of JIAD, especially in precise harvesting and shaping of the fibular flap and prosthetic rehabilitation using dental implants. While multiple studies pertaining to fibular flaps have been documented [4, 8, 9], the bone volume and height of the fibular flap may be insufficient for the placement of dental implants. The presence of a considerable amount of bone makes an iliac flap the ideal choice in these cases. Here, we present the first case report to document the use of one-stage jaw reconstruction and prosthetic rehabilitation with the iliac flap. In this case report, we document two cases of mandibular reconstruction performed using the iliac flap and immediate implant-retained prosthesis, which was planned using VSP. We modified the original workflow for use of the iliac flap.

## Case presentation

### Virtual surgical planning

Preoperative computed tomography scans of the head, neck, and ilium region of the patient were used for VSP. Tumor resection was simulated using ProPlan CMF 3.0 (Materialise, Belgium). The defect was reconstructed by mirroring the unaffected side, and the ilium of the side corresponding to the mirrored side of the mandible was selected for reconstruction. The iliac crest was positioned upward to provide cortical bone for achieving the primary stability of dental implants. The iliac bone was placed 12 to 15 mm below the occlusal plane to create adequate space for the implant-retained prosthesis. Three models were 3D printed: those of the reconstructed mandible, defective mandible, and iliac bone. A 2.0-mm reconstruction plate (DePuy Synthes, USA) was pre-bent by the surgeons 3 days before the surgery, according to the contour of the reconstructed mandibular model. The mandibular and iliac surgical guides were designed according to a previous study [10]. The dental implant prosthesis was designed, and the positions of dental implants were determined using 3Shape Implant Studio (3Shape, Denmark). All guides and prostheses were fabricated using computer-aided design and computer-aided manufacturing.

### Surgical procedure

Mandibulectomy was performed using the mandibular surgical guide. The reconstruction plate was fixed in the predetermined site [10]. The iliac model was used to determine the range of osteotomy. An iliac flap was harvested and shaped using the iliac surgical guide.

**Fig. 1:**
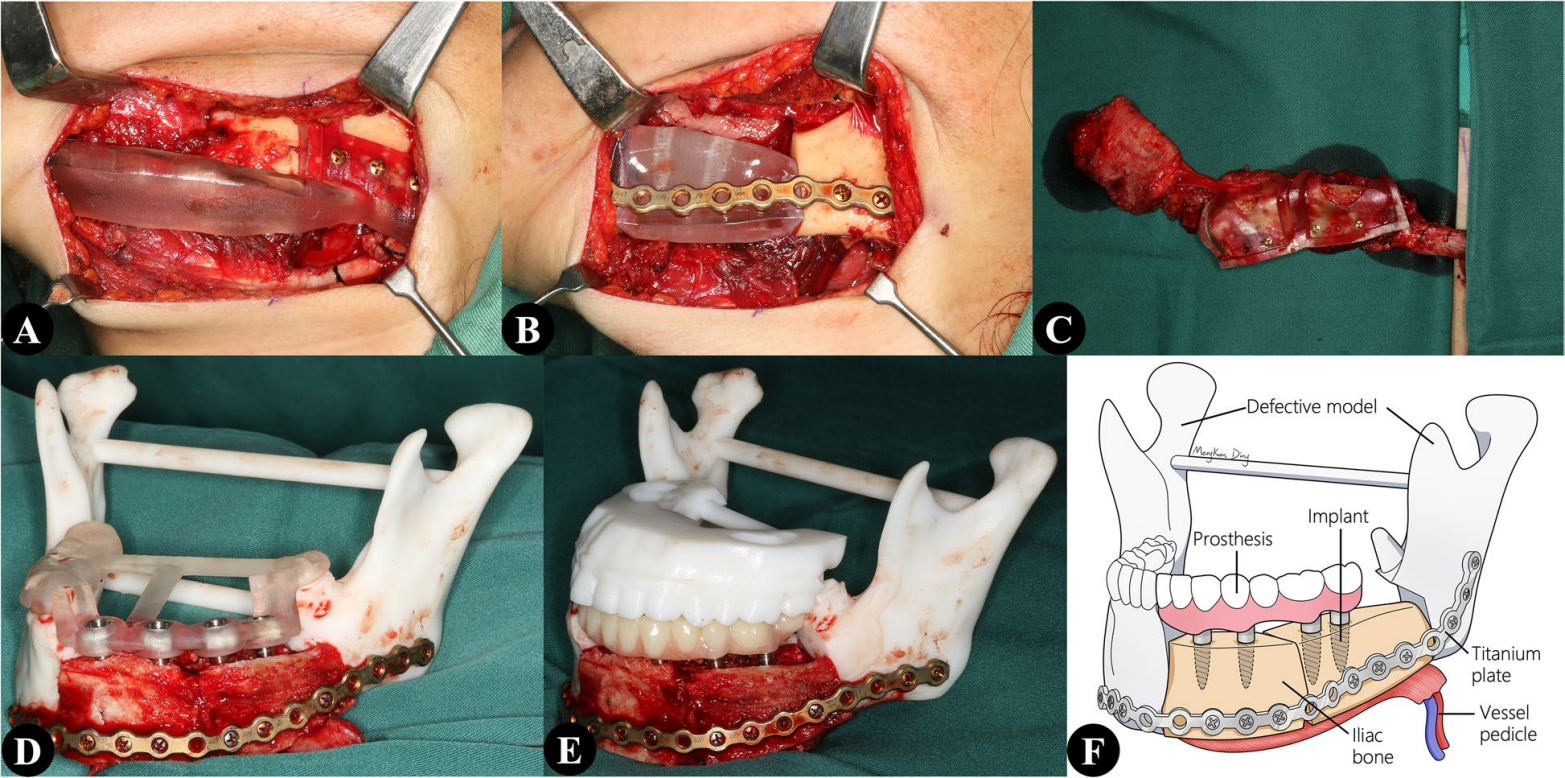
**A** Mandibulectomy performed using a mandibular surgical guide. **B** The iliac model used to determine the range of osteotomy. **C** An iliac flap harvested and shaped using the surgical guide. **D** After securing the iliac flap in the defective mandible model, an independent implant guide used for implant insertion. **E** Assessment of the occlusal relationship after prosthesis delivery. **F** The details of prosthesis-implant-flap-plate complex

Before cutting off the vascular pedicle, the iliac flap was fixed in the defective mandibular model using the pre-bent reconstruction plate. The implant guide was attached to the defective mandibular model, and dental implant insertion was performed. Individual abutments were connected for the fabrication of the implant prothesis using a resin adhesive. A maxillary model was also 3D printed for verifying the occlusion in vitro. In the last step, the prosthesis, implants, iliac flap, and reconstruction plate became a fixed complex, which was transferred to the recipient site as a whole (Fig. 1).

After vascular anastomosis, the complex was fixed to the residual mandible. Checking the occlusal relationship is an extremely important step to avoid any premature contacts between the mandibular implant prosthesis and maxillary teeth, which can affect the healing of the iliac bone negatively. Occlusal adjustment was performed if necessary. Primary closure over the bone was achieved underneath the prosthesis. The best choice for intraoral wound closure is the original gingiva. Otherwise, the fascial tissue of the iliac flap is also acceptable.

### Postoperative management

In the first postoperative week, patients were given a liquid diet through a nasal feeding tube. The liquid diet is advised until 1 month after the surgery. Thereafter, a soft diet is recommended, such as porridge, congee, and puree. Patients were advised to resume routine mastication and normal diet 3 months after the surgery. Cone-beam computed tomography scans were obtained 3, 6, and 12 months postoperatively to assess the condition of dental implants and bone union. The provisional prosthesis was replaced by a final prosthesis.

### Case presentation

**Fig. 2:**
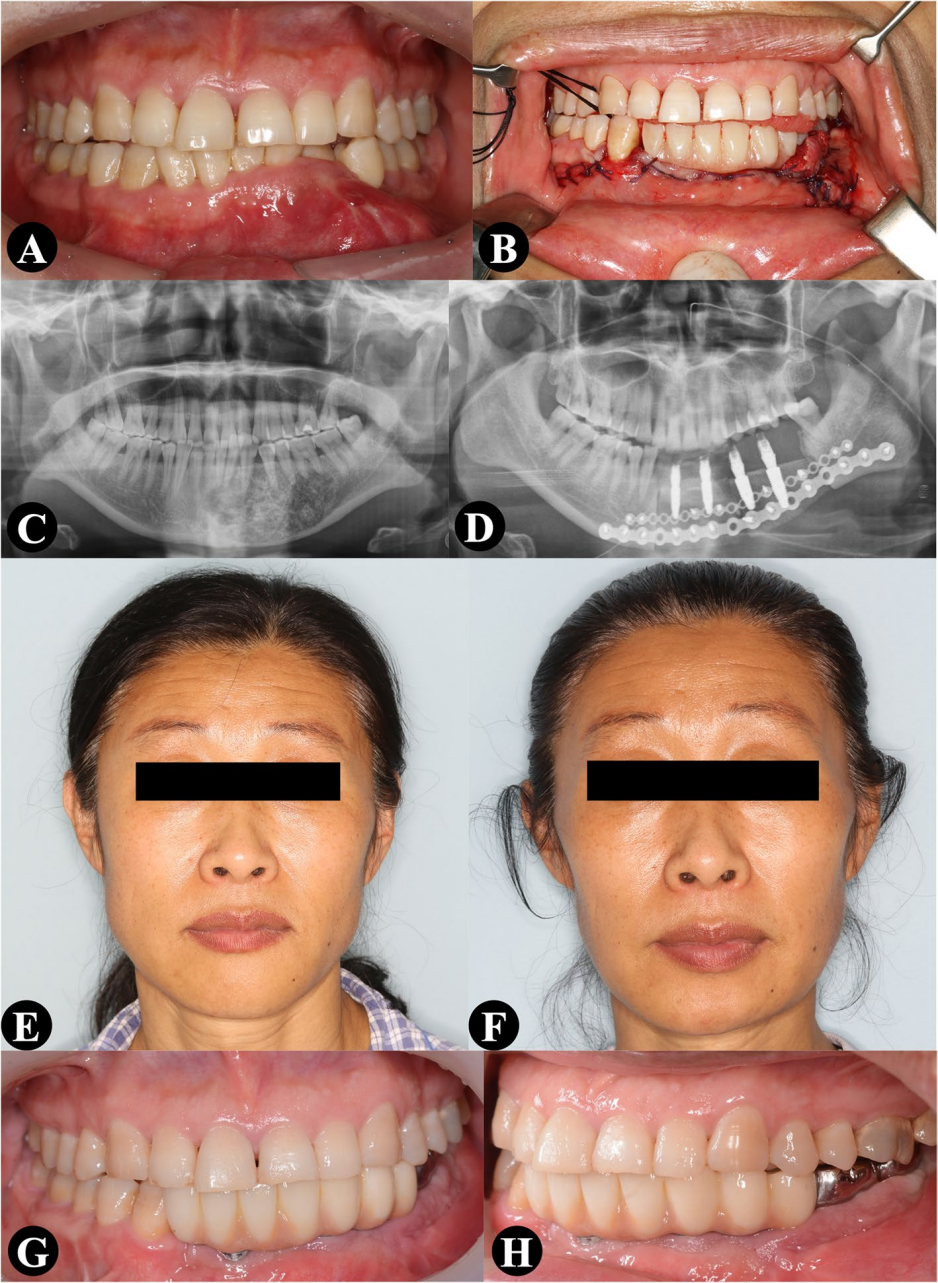
A 45-year-old woman presented with a left mandibular ameloblastoma. The mandibular defect was reconstructed using a two-segment iliac flap. Four dental implants were inserted, and an immediate implant-retained prosthesis was delivered. **A** and **B** Preoperative and postoperative intraoral profile. **C** and **D** Preoperative and postoperative panoramic radiograph. **E** and **F** Preoperative and postoperative facial profile. **G** and **H** Final intraoral profile with the definitive dental prosthesis

We present two cases in this report. A left mandibular tumor was observed in the orthopantogram of a 45-year-old woman. Histopathological analysis revealed an ameloblastoma. After a multidisciplinary discussion, one-stage mandibular reconstruction was planned. Several customized surgical guides were fabricated. The mandibular defect was 5.05 cm, and the mandible was reconstructed using a two-segment iliac flap. Four dental implants were inserted, and an immediate implant-retained prosthesis was delivered. The implants and prosthesis were in function when they were evaluated after a year. The follow-up duration was 29 months, and no short-term or long-term complications occurred (Fig. 2).

**Fig. 3:**
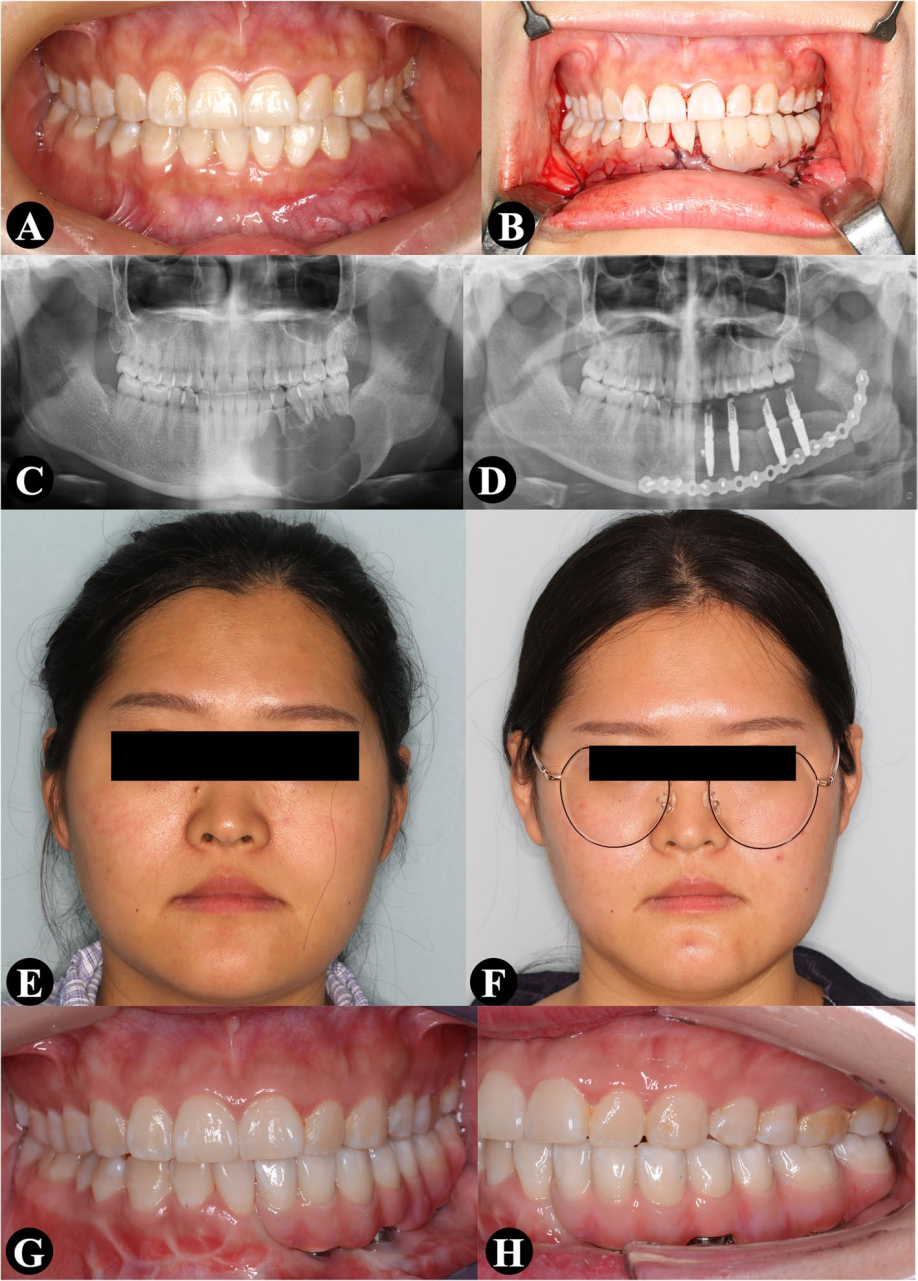
A 24-year-old woman presented with a left mandibular ameloblastoma. One-stage mandibular reconstruction was performed using a two-segment iliac flap. Four dental implants were inserted, and an immediate implant-retained prosthesis was delivered. **A** and **B** Preoperative and postoperative intraoral profile. **C** and **D** Preoperative and postoperative panoramic radiograph. **E** and **F** Preoperative and postoperative facial profile. **G** and **H** Final intraoral profile with the definitive dental prosthesis

A 24-year-old woman presented with a tumor in the left mandible that she had for 4 years. Histopathological analysis revealed an ameloblastoma. The defect was 6.43 cm after mandibulectomy. As described above, one-stage mandibular reconstruction was performed using a two-segment iliac flap. Four dental implants were inserted, and an immediate implant-retained prosthesis was delivered. The implants and prosthesis were in function when they were evaluated after a year. Peri-implantitis was evident with the mesial second implant after 1 year of follow-up. Free gingival graft (FGG) was used to improve the peri-implant condition. Bone level was stable until 20 months postoperatively (Fig. 3).

## Discussion

The JIAD technique was introduced by Levine et al. in 2013. [8] Both mandibular reconstruction and prosthetic rehabilitation using dental implants can be finished at the same stage in 1 day by using the JIAD technique. In our clinical experience, five key points are crucial for the success of the JIAD technique. First, preoperative VSP is the foundation for the JIAD technique. Second, occlusion-driven jaw reconstruction needs multidisciplinary cooperation among a team comprising at least oral and maxillofacial surgeons, prosthodontists, and dental laboratory technicians. Third, the dental prosthesis, implants, iliac flap, and reconstruction plate should be a fixed complex when transferred to the recipient site. Fourth, the original gingiva should be preserved to the maximum extent especially in cases with complete tumor resection. Lastly, periodontal maintenance therapy is extremely important for these patients to achieve a good long-term result.

The fibular flap became the first choice of flap in most cases of mandibular reconstruction in the past decade [11]. In the previous studies, the fibular flap was used in all cases (Table 1) [4, 8, 12–15]. The main disadvantage of the fibular flap is that the lack of height of fibular bone made dental implant insertion less convenient [16]. The iliac flap can provide a greater amount of bone, so that 14 mm or longer implants can be used to achieve a good long-term result. We also placed the iliac crest facing upward to reconstruct the alveolar crest instead of the mandibular lower margin, which is a technique different from that used in previous studies. The cortical bone of the iliac crest can help in achieving adequate primary stability for the immediately loaded implants.

**Table 1:** Cases published in the literature of recorded “jaw-in-a-day” procedures

Reference	Year	*n*	Gender	Average age (year)	Pathology	Location	Flap	Average implants	Average teeth restoration	Follow-up time (month)
Levine et al. [8]	2013	4	2 M, 2 F	25.8 (20–34)	2 Ame, 1 OM, 1 AM	3 Man, 1 Max	Fibular flap	5.3 (4–6)	8.8 (4–12)	14.5 (10–22)
Qaisi et al. [12]	2016	3	2 M, 1 F	44.3 (23–76)	3 Ame	3 Man	Fibular flap	3.7 (3–4)	7.0 (5–10)	11.0 (4–17)
Runyan et al. [13]	2016	1	F	26.0	ES	Max	Fibular flap	3.0	8.0	5.0
Patel et al. [4]	2019	2	2 F	19.5 (19–20)	2O M	1 Man, 1 Max	Fibular flap	3.5 (3–4)	6.5 (5–8)	NA
Sukato et al. [14]	2020	2	1 M, 1 F	43.0 (35–51)	1 Ame, 1 Tra	2 Man	Fibular flap	5.5 (5–6)	12.0 (12–12)	6.0 (6–6)
Grewal et al. [15]	2021	1	M	51.0	OKC	Man	Fibular flap	3.0	10.0	24.0

*Abbreviations*: *M*, male; *F*, female; *Ame*, ameloblastoma; *OM*, odontogenic myxoma; *AM*, arteriovenous malformation; *ES*, Ewing’s sarcoma; *Tra*, trauma; *OKC*, odontogenic keratocyst; *Man*, mandible; *Max*, maxilla; *NA*, not available

In the conventional approach of two-stage mandibular reconstruction, implants are inserted in the reconstructed mandible at least 6 months postoperatively. However, the postoperative anatomy might not be suitable for implant-retained prosthesis because of deficient bone, malpositioned bone, or bulky soft tissues [17]. In such cases, multiple surgeries are required to improve the peri-implant condition, delaying the time of prosthesis insertion. Some studies showed that the maximum delay in prosthesis insertion using the traditional approach was 12 to 18 months [4, 8]. The long “edentulous period” can have severe psychological sequelae in such patients. The JIAD technique can help in reducing the treatment duration significantly [8]. It can also allow us to eliminate the time of partial or total edentulism, aiding in patients’ recovery both functionally and psychologically [8].

However, the JIAD technique is associated with some challenges. Previous authors have used skin grafts and carbon dioxide laser to reduce the possibility of peri-implant mucositis and peri-implantitis [14]. Of our two cases, peri-implantitis occurred in one patient, and FGG was used to improve the peri-implant condition. Chang et al. believed that the use of the JIAD technique needed more time for preoperative preparation and more economic investment for the fabrication of customized surgical devices. [9] They also highlighted a disadvantage that the treatment plan cannot be changed during the surgery [9]. Therefore, they used a pre-bent reconstruction plate instead of a 3D-printed plate, and the prosthesis was designed and fabricated after fibular bone fixation and dental implant insertion [9]. We also used a pre-bent reconstruction plate, which is time- and cost-efficient, though the accuracy of 3D-printed plates is higher than that of pre-bent plates.

In previous studies, fibular surgical guides were designed for both fibular osteotomy and implant placement. However, some factors that could cause changes in implant positions, such as the soft tissues of the flap preventing the seating and leading to sliding of the surgical guides, were evident [18]. Moreover, the shaping of multiple segments of the DCIA flap is more difficult than that of the fibular flap. To prevent the effect of shaping errors of iliac bone and malpositioning of surgical guides on implant placement, we used an independent implant surgical guide. In our workflow, even if the shaping of iliac bone deviates from the planned shape, the relative position between the implants and the residual mandible does not change, which can achieve a passive fit and finish of the prosthesis.

In our clinical practice, the JIAD technique is performed only in patients with benign tumors. Only one case was malignant tumor in previous studies (Table 1) [13]. There are several reasons. First, patients with malignant tumors may require postoperative radiotherapy, which can negatively affect the long-term outcomes of implants [19]. Second, several customized surgical devices need to be fabricated preoperatively, requiring a prolonged preoperative preparation time and delay in the surgery. Third, resection of malignant tumors may lead to soft-tissue defects, especially gingival defects, which may negatively affect the long-term outcomes of implants [20].

## Conclusion

To the best of our knowledge, this is the first report to document the use of the JIAD technique with the iliac flap for mandibular reconstruction. The iliac flap might be another choice for mandibular reconstruction using the JIAD technique, especially for benign tumor.

### Supplementary Information

**Additional file 1.**

## Data Availability

Not applicable.
