# Supplementary figures and images for: One-stage jaw reconstruction and prosthetic rehabilitation with an iliac flap: a case report and literature review
Source: Maxillofac Plast Reconstr Surg. 2024 Jan 17;46(1):3. doi: 10.1186/s40902-024-00413-0 (PMC10794675; doi:10.1186/s40902-024-00413-0)

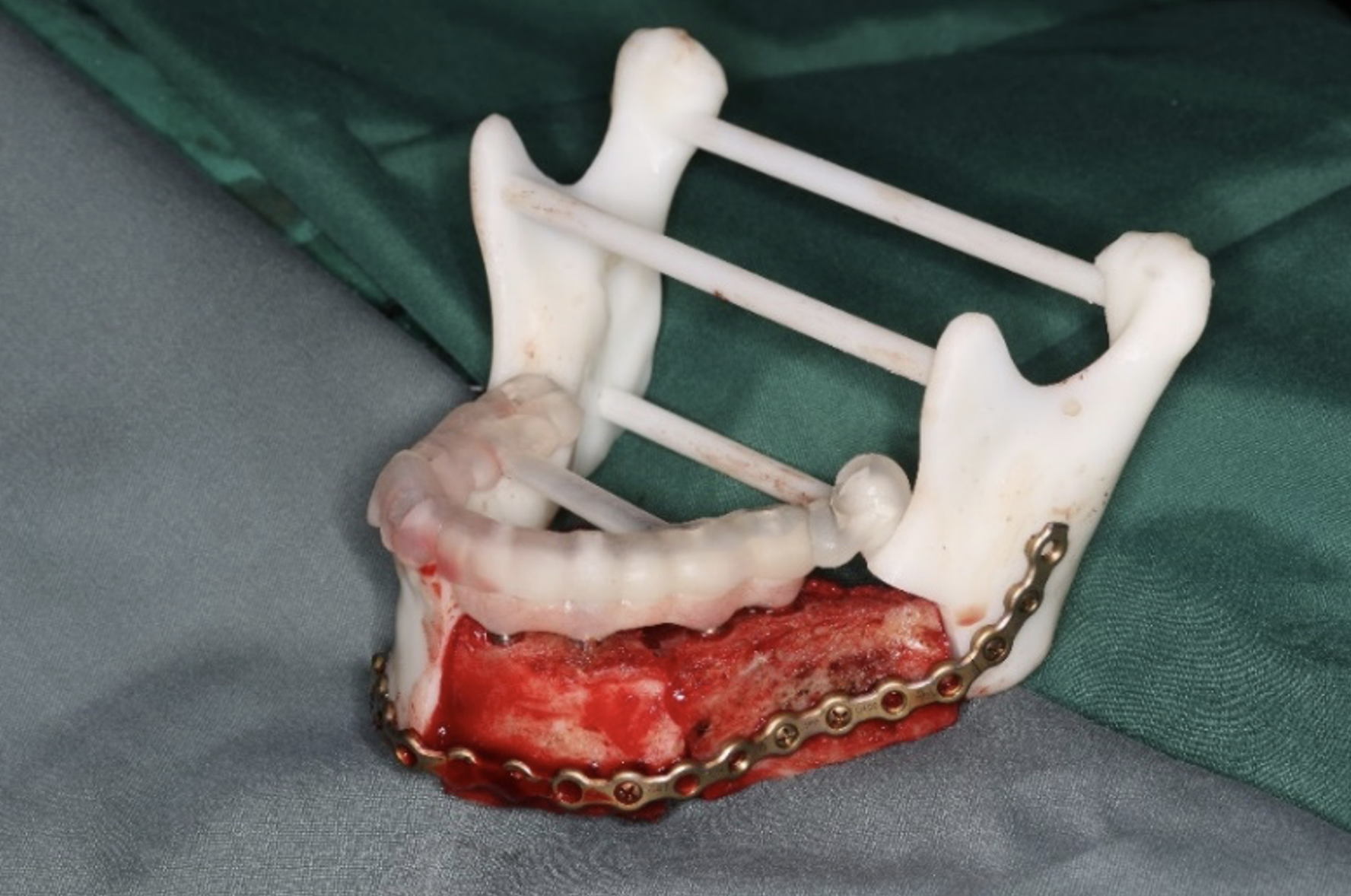

Supplement: Supplementary file 1 — Additional file 1. [file 40902_2024_413_MOESM1_ESM.png]
